# Managing traumatic brain injury secondary to explosions

**DOI:** 10.4103/0974-2700.62120

**Published:** 2010

**Authors:** Paula Burgess, Ernest E Sullivent, Scott M Sasser, Marlena M Wald, Eric Ossmann, Vikas Kapil

**Affiliations:** National Center for Environmental Health, Centers for Disease Control and Prevention, USA; 1 Division of Injury Response, National Center for Injury Prevention and Control, Centers for Disease Control and Prevention, USA; 2 Department of Emergency Medicine, Emory University School of Medicine, USA; 3 Department of Emergency Medicine, Duke University School of Medicine, USA

**Keywords:** Explosions and bombings, traumatic brain injury, wounds and injuries

## Abstract

Explosions and bombings are the most common deliberate cause of disasters with large numbers of casualties. Despite this fact, disaster medical response training has traditionally focused on the management of injuries following natural disasters and terrorist attacks with biological, chemical, and nuclear agents. The following article is a clinical primer for physicians regarding traumatic brain injury (TBI) caused by explosions and bombings. The history, physics, and treatment of TBI are outlined.

## INTRODUCTION

Disaster medical response training has traditionally focused on the management of injuries following natural disasters and terrorist attacks involving biological, chemical, and nuclear agents. However, explosions and bombings remain the most common deliberate cause of disasters involving a large number of casualties, leading some experts to consider blasts or explosions as the ‘fourth weapon of mass destruction.’[1] Despite the ever-present threat of terrorist bombings in the US, the civilian medical community lacks significant training and experience in the diagnosis and treatment of blast injury.[1]

There are an average of five criminal bombing incidents per day in the United States. Major domestic terrorist bombings include events at the World Trade Center (New York City) in 1993, the Frank L. Murrah Federal Building (Oklahoma City) in 1995, and the Olympic Park bombing (Atlanta) in 1996.[2] Yet, most US healthcare providers have considered the diagnosis and management of blast injuries to be more of a problem for other countries rather than the United States, and few have extensive training in the management of these complex casualties.[3]

Severe head trauma has been identified as a common cause of death in terrorist bombings and has been found to be a major cause of critical injury in a number of these events.[4,5] During Operation Iraqi Freedom, explosive munitions were found to be the most common primary causative agent for the injuries in soldiers wounded in action.[6] Additionally, data from Operation Iraqi Freedom and Operation Enduring Freedom also demonstrate an increasing incidence of head and neck wounds, with a concomitant increase in brain injury, when compared to previous conflicts.[7]

Blast-associated traumatic brain injury (TBI) is a condition that requires rapid recognition and therapy. The time-critical nature of this condition requires that acute care providers become familiar with the pathophysiology, diagnosis, and treatment considerations for blast-associated TBI in advance of an explosive event. It is also important for acute care providers to recognize and understand the long-term sequelae of blast-associated TBI to insure appropriate referral and follow-up.

## DEFINITIONS

Potential central nervous system sequelae of bomb blast injury include concussion, post-concussion syndrome (PCS), mild traumatic brain injury (MTBI), post-traumatic stress disorder (PTSD), and acute stress disorder (ASD). Definitions of these terms vary and the sign–symptom complexes overlap, adding to the confusion and uncertainty regarding the nature of brain injuries following exposure to blast effects. Hence, it is important to address the definitions and relationships among these entities [Table 1].

**Table 1:** Inclusion criteria for a diagnosis of mild traumatic brain injury

**Organizational source for definition**	**Inclusion criteria**
American Congress of Rehabilitation Medicine	1. Any period of loss of consciousness (LOC) of less than 30 min and a Glasgow Coma Scale (GCS) score of 13-15 after the period of LOC.
	2. Any loss of memory of the event immediately before or after the injury with post-traumatic amnesia (PTA) of less than 24 h.
	3. Any alteration in mental state at the time of the injury (e.g., feeling dazed, disoriented, confused).[8,9]
Centers for Disease Control and Prevention (CDC)	1. Any period of observed or self-reported transient confusion, disorientation, or impaired consciousness.
	2. Any period of observed or self-reported dysfunction of memory (amnesia) around the time of injury.
	3. Observed signs of other neurologic or neuropsychological dysfunction.
	4. Any period of observed or self-reported loss of consciousness lasting 30 min or less.[10]

The Defense and Veterans’ Brain Injury Center (DVBIC) Working Group on the Acute Management of MTBI in Military Operational Settings defines MTBI as an injury to the brain resulting from an external force and/or acceleration/deceleration mechanism from an event such as a blast, fall, direct impact, or motor vehicle accident, which causes an alteration in mental status that typically results in the temporally related onset of symptoms such as headache, nausea, vomiting, dizziness/balance problems, fatigue, insomnia/sleep disturbances, drowsiness, sensitivity to light/noise, blurred vision, difficulty remembering, and/or difficulty concentrating.[11]

As defined by the American Academy of Neurology (AAN), concussion is a trauma-induced alteration in mental status that may or may not involve loss of consciousness.[12] Mild forms of concussion can manifest as a sensation of being dazed or ‘starstruck,’ and loss of consciousness is not required.[13] Although the AAN definition does not state this explicitly, some clinical experts consider that concussion effects are transient and MTBI effects are either semi-permanent or permanent; other experts use the terms interchangeably.[8]

PCS refers to the cluster of symptoms that frequently occur in the days or weeks following an MTBI and include headache, dizziness, irritability, difficulty concentrating, memory problems, fatigue, visual disturbances, sensitivity to noise, judgment problems, depression, and anxiety. Although PCS often resolves within a month, it is not uncommon for individuals to experience a persistent PCS, with symptoms lasting months to years after an MTBI.[14]

PTSD is a psychological disorder that was first formally recognized in the third edition of the *Diagnostic and Statistical Manual of Mental Disorders (DSM-III)* in 1980. Since then, PTSD has been used in both the civilian and military populations to describe a symptom complex that occurs after exposure to a psychologically traumatizing event and lasts for longer than 1 month. Symptoms include mood lability, agitation, anxiety, depression, fatigue, and nightmares.[15] ASD generally refers to a similar constellation of signs and symptoms that lasts from 2 days to 4 weeks.[16]

One Veterans’ Administration Hospital study of combat veterans with chronic PTSD noted conclusive and statistically significant discriminate electroencephalography (EEG) evidence of MTBI in a subgroup of veterans who reported a history of blast-related concussion from World War II, the Korean War, the Vietnam conflict, or the First Gulf War as compared to veterans with no history of blast-related concussion.[17] Furthermore, greater than half of those in the blast-injured group without preinjury evidence of attention-deficit hyperactivity disorder (ADHD) met the criteria for this disorder, exhibiting problems with attention, impulsivity, or hyperactivity that interfered with daily function.[17]

In a large study of patients injured by explosive munitions, 200 (30%) of 665 of those with extremity wounds had, in addition to signs and symptoms consistent with primary blast injury, long-standing (1 year) signs and symptoms reflecting central nervous system disorders compared to only 4% of patients without blast trauma.[18]

These studies suggest that some patients diagnosed with combat-related PTSD may also have features of blast-related PCS or MTBI. Identification of individuals who have MTBI from within the PTSD group has important clinical implications in terms of treatment and long-term prognosis.[17]

## BLAST INJURY: A HISTORICAL PERSPECTIVE ON ‘SHELL SHOCK’ AND TBI

A historical perspective on blast injury to the brain during World War I is relevant to today's sometimes confusing discussions of concussion, PCS, MTBI, and PTSD.[15] The impact of blast injury on human beings was first described during World War I by British physicians in field hospitals.[19] The physical and psychological conditions they observed in survivors of blasts came to be known as ‘shell shock.’[20,21] In this diagnosis, a variety of physiological and neuropsychiatric symptoms were characterized among those individuals who had been in proximity to explosions; these symptoms included cognitive and memory impairment, lability of mood and other mood disorders, and attention and/or hyperactivity disorders[19] Based on clinical battlefield experiences, Major F.W. Mott, a surgeon in the British Expeditionary Force, delivered a series of lectures in London referring to concussion, or ‘*commotio cerebri*’ produced by ‘aerial compression.’[22] His published lectures spurred others to provide written case reports describing similar neuropsychiatric symptoms in soldiers presenting for post-blast treatment at the front.[21] Such reports persuaded the Army Council of Britain to classify shell shock as a battle wound late in 1915 and to recommend rapid evacuation of affected soldiers.[21] The controversy regarding physical *vs* psychological causes for shell shock, which was also termed ‘cerebral blast syndrome’ and ‘cerebral blast concussion,’ continued into World War II,[23,24] and is even reflected today in the differing opinions expressed by researchers and clinicians.

## EPIDEMIOLOGY OF BRAIN INJURY: CIVILIAN TERRORIST BOMBINGS AND MILITARY EXPERIENCE

Severe head trauma is a common cause of death in terrorist bombings. An analysis of 14 published studies of terrorist bombing incidents involving 3357 documented casualties between 1969 and 1983 demonstrated that head injury was a common cause of both immediate fatality and late fatality.[5] Additionally, postmortem findings from the 1983 bombing of the Marine barracks in Beirut revealed that wounds to the head were the most common cause of immediate (71%) and late fatalities (52%).[5] The incidence of head injury in the 1995 Oklahoma City bombing was 14%.[25] In the attack on the USS Cole in 2000, the incidence of head injury was 31%.[26] In the 2004 Madrid train bombings, 12% of the 250 injured that were treated at the closest hospital had head injury; among the 29 critically injured, 52% suffered head trauma.[4]

The epidemiology of TBI in the military has changed with the advent of the use of body armor. The effectiveness of body armor may contribute to a higher incidence of TBI in survivors because there is decreased mortality secondary to torso wounds. Many individuals who would have died in previous conflicts now survive; among those who do survive, a greater percentage have brain injuries.[27] The use of Kevlar™ helmets has greatly reduced the incidence of penetrating head injuries from projectiles, but the brain remains susceptible to concussive forces.[28] Furthermore, the increased use of improvised explosive devices (IEDs) as weapons has contributed significantly to the incidence of TBI.[29]

During the First Gulf War in 1991, about 20% of those treated for wounds had head injuries.[27] In the current conflicts in Iraq and Afghanistan, blasts are the most common cause of wounds and the leading cause of TBI.[27] Approximately two-thirds of army war zone medical evacuations are due to blast injury, and 88% of second echelon treatment site injuries are due to blast trauma.[27] Since the beginning of the current conflicts, over 1700 individuals have sustained TBIs. A descriptive analysis of 433 individuals with TBI seen at the Walter Reed Army Medical Center indicates that MTBI accounted for less than half of the sample; moderate and severe (including penetrating) brain injury accounted for 56%. Penetrating brain injury accounted for 12% of the total group, while closed TBI accounted for 88%. The number of those with serious brain injury has been estimated to exceed those with amputations by 500%, which is in marked contrast to the pattern of wounds in World War I and World War II.[27] At a regional Veterans’ Administration hospital, the number of TBI admissions almost doubled over a 2-year period prior to June 2005.[28]

In critically reviewing the literature, it is important to note the difference between ‘head injury’ and ‘TBI.’ A head injury is an injury to the head that is clinically evident on physical examination and is recognized by the presence of physical findings such as ecchymoses, lacerations, deformities, or cerebrospinal fluid leakage. This head injury may or may not be associated with brain injury. In contrast, a ‘TBI’ specifically refers to an injury to the brain itself and is not always immediately clinically apparent. Some epidemiological studies of injury patterns make no differentiation between these two entities, making it difficult to determine the true epidemiology of trauma to the brain.[30]

Accurate estimates of the incidence of MTBI is also challenging due to the lack of agreement on its definition and diagnostic criteria. An increased awareness of MTBI may account for some increase in reported incidence in recent years. However, it is likely that underdiagnosis of MTBI following explosive events mimics the underdiagnosis in the area of sports concussions. Many with MTBI never even seek medical care and therefore are not counted as cases. The frequency with which MTBI is undiagnosed has resulted in the terms ‘secret epidemic,’ ‘silent epidemic,’ or ‘silent injury.’[31]

One study of abstracted medical records of survivors treated at 36 New York City hospitals following the World Trade Center attacks on September 11, 2001, demonstrated that 21 (60%) out of 35 cases retrospectively diagnosed as probable TBI (mostly from falling debris) were undiagnosed during the hospital stay.[32] In terrorist bombings, it is reasonable to assume that TBIs may similarly be underdiagnosed. In war as well as in the civilian terrorist bombing events, MTBI from explosions likely goes undiagnosed when those injured sustain other acute and life-threatening injuries. In addition, it is possible that some patients who receive a diagnosis of PTSD after exposure to a blast event may actually have PCS and MTBI, another potential cause for underestimation of MTBI incidence.

## BLAST PHYSICS

The physics of explosions and blast waves are complex and nonlinear. Understanding some of the basic principles can provide clinicians with insight into possible injuries sustained from explosive events and lead to more accurate diagnosis and improved outcomes. Explosions are the result of rapid chemical conversion of solids or liquids into gases, with an ensuing energy release in the form of a blast overpressure wave and blast wind. Initially and transiently, these gases occupy the same volume as the parent solid or liquid, creating an extremely high pressure. These gases then expand rapidly, compressing the surrounding air and creating the positive overpressurization phase or the positive phase impulse of the blast wave.[33] The term ‘shock wave’ has been used to describe this abrupt, virtually instantaneous, increase in pressure from high-explosive blast waves. The shock waves resulting from high-energy explosions possess a shattering effect, which is termed ‘brisance.’ The magnitude of the blast injury is dependent upon the magnitude of the positive phase impulse.[34] Shock pressure waves of 5 pounds per square inch (PSI) or more can cause eardrum rupture; 16 PSI is enough to cause lung injury; and 30-42 PSI is the threshold pressure for lethality.[35] Building collapse may occur with overpressure as low as 10 PSI. For comparison, even exceedingly loud acoustic waves cause overpressurization of less that 0.04 PSI.[34]

As the gas created by the explosion expands rapidly into a larger space, a relative vacuum is created behind the wave, and the overpressurization phase is followed by a temporary underpressurization phase of the blast wave. When a blast wave impacts the body, relatively high-frequency ‘stress waves’ and relatively low-frequency ‘shear waves’ are created. Stress waves result from rapid acceleration of the body surface. Shear waves are created by the magnitude of body-wall displacement and are generally perpendicular to stress waves and tangential to the body surface.[33] Injury sustained as a result of the impact of the overpressurization wave is termed *primary* blast injury.

In addition to these blast wave forces, blast winds are also created in explosions. Blast winds move away from the epicenter of the explosions during the positive pressure phase of the blast, but quickly reverse and move back toward the epicenter during the negative pressurization phase of the blast wave.[33] Blast winds can exceed hurricane force winds. These wind velocities can be magnified substantially by external conditions, e.g., by channeling through corridors or alleys.[34]

Other external conditions that significantly affect blast physics, and therefore blast injuries, are distance from the epicenter and physical features of the space where the blast occurs. Peak overpressures in an open space explosion are directly related to the energy of the blast and inversely proportional to the cube of the distance from the epicenter. Thus, increased distance from the epicenter of an explosion implies decreased likelihood, and a lesser severity, of primary blast injuries.[34]

Victims who are in enclosed spaces at the time of the blast have the highest proportion of primary blast injuries after detonations, regardless of whether the explosion occurred inside or outside the enclosure. Solid surfaces (e.g., walls, floors, ceilings, etc.) can reflect blast waves, creating more intensely pressurized air. Individuals near walls are potentially subjected to exponentially increased overpressurization by wave reflection and those in corner areas of buildings even more so.[34]

Explosions that occur underwater alter blast physics in such a way that greater blast loading is exerted on the most deeply submerged parts of a victim. The increased velocity of blast waves in water extends the lethality of underwater shock waves approximately three times farther than equivalent detonations in open air.[34]

Blast physics also depend upon whether low-order or high-order explosives are involved. Propellants (e.g., gunpowder, pipe bombs, etc.) are low-order explosives designed to release energy relatively slowly by a burning process called ‘deflagration.’[34] The blast waves resulting from this process are subsonic. In contrast, high-order explosives (e.g., trinitrotoluene [TNT], Composition 4, Semtex^®^, nitroglycerin, dynamite, ammonium nitrate fuel oil [ANFO], etc.) create high-energy supersonic blast waves upon detonation. In general, shear waves predominate in higher-order explosive events and stress waves predominate in lower-energy events.[34] High-order explosives are necessary for the production of the overpressurization wave. The blast wind is a consequence of both high-order and low-order explosives. The majority of blast injuries are the result of high-order explosives.[36,37]

The severity of blast injuries may be augmented by the addition of certain materials to the bomb. Incendiary agents (e.g., napalm) increase the adherence of flammable products and prolong burning time, resulting in greater burn injuries. Bombs can be designed to release noxious chemical, radiological, and biological agents upon detonation. Metallic objects (e.g., nails, screws, etc.) can be added to IEDs to increase the number of penetrating injuries,[38] including penetrating brain injury.

## MECHANISMS AND MANIFESTATIONS OF TBI EXPLOSION INJURIES

Blast injuries are categorized by primary, secondary, tertiary, and quaternary effects. *Primary* blast injuries are those caused by barotrauma from the overpressurization blast wave. Generally, the parts of the body most susceptible to primary blast injury are air-filled organs and body areas with air–fluid interfaces. Ruptured tympanic membrane and blast lung injury are examples of primary blast injuries. *Secondary* blast injuries result from penetrating or blunt trauma by projectiles hurled as a consequence of blast waves or blast wind. *Tertiary* blast injuries result from forceful displacement of the body by the blast wind and any resulting impact against obstacles. *Quaternary* injuries are those injuries related to explosions, but not caused by either primary, secondary, or tertiary mechanisms. Examples include burns, toxic inhalations, radiation injury, chemical exposures, as well as exacerbations or complications of preexisting conditions (e.g., exacerbation of asthma, myocardial infarction).[37] Injuries resulting from structural collapse and fallen debris, such as crush injuries and compartment syndrome, are also examples of quaternary injury.[1] *Quinary* effects have also been postulated. In the quinary mechanism of blast injury, a hyperinflammatory, hyperpyrexic body reaction is induced by the toxicity of some unconventional explosive materials utilized in the bomb, (e.g., some fertilizers).[39]

The presence of bony protuberances on the inferior cranial vault, as well as the delicate composition of the cerebral cortex, brainstem, and axonal fibers, render the brain especially vulnerable to injury.[28] The most common types of nonpenetrating TBI are diffuse axonal injury, cerebral contusion, and subdural hematoma.[33] Diffuse axonal injuries are common following closed head injuries and result from shearing forces pulling on neuronal axons and small vessels in the brain. Cerebral contusions occur due to movement of the brain within the skull, such as in coup-contrecoup injuries. Subdural hematoma results from movement of the brain within the skull, with tearing of the bridging veins between the brain surface and the dural venous sinuses.[28] Both open and closed skull fractures can occur. A blunt force over the temporal area can cause skull fracture and subsequent middle cerebral artery bleeding, with resulting epidural hematoma. These pathological findings of brain injury can result in a spectrum of acute clinical presentations, ranging from mild concussion to focal neurological findings, unconsciousness, coma, and death.

While secondary and tertiary blast mechanisms result in blunt or penetrating trauma similar to head injury from causes other than explosions, a discussion of primary blast injury to the brain is more complicated and controversial. One mechanism of primary blast injury is arterial air embolism to the central nervous system as a consequence of alveolar damage in blast lung injury. These air emboli are life threatening and can cause temporary unconsciousness or focal neurological complaints similar to that seen in a stroke.[40] An extreme example of brain injury suffered as a consequence of primary blast effects is documented in studies that demonstrate that stress waves and blast winds can also be of sufficient magnitude to shatter the skull or result in decapitation.[34,41–43]

Researchers do not agree whether primary blast effects cause nonfatal brain injuries. While some say the homogeneous nature of the brain makes this unlikely,[34] others suggest that deceleration and shearing can occur due to the differing densities of gray and white matter, resulting in diffuse axonal injury.[44] Rat and rabbit animal models have unequivocally demonstrated the vulnerability of the brain to primary blast forces. In these studies, the animals were protected against any secondary and tertiary blast injuries by strapping and cushioning. Exposure to an overpressurization wave caused microscopic, biochemical, and clinical evidence of central nervous system injury.[45–49]

## CLINICAL PRESENTATION AND DIAGNOSIS

The clinical presentation of survivors with TBI from explosion is similar to those with open or closed brain injuries due to other mechanisms, and the approach to diagnosis remains the same. The diagnosis of penetrating head wounds is generally as straightforward as the presentation is dramatic. Diagnosis of open and closed skull fractures, concussions, contusions, and subarachnoid, subdural, and epidural hematomas presents the same challenges in survivors of bombings as in those injured by other mechanisms.

In the diagnosis of TBI resulting from explosion, the first step is a clinical assessment that includes a history of the mechanism of injury, context of the event, and presence or absence of periods of loss of consciousness and posttraumatic amnesia (PTA). In the unconscious or uncommunicative patient, a history should be obtained from bystanders or prehospital personnel, if possible. The environment of the bombing (e.g., open space *vs* closed space, proximity of victim to bomb, etc.) can alert the acute care clinician to the possibility of primary blast injury.

The Glasgow Coma Scale (GCS) can be a useful tool in evaluating central nervous system (CNS) function.[50] Serial scoring, especially in the prehospital setting, can identify CNS deterioration that may be indicative of the need for neurosurgical intervention. A low initial GCS that remains low, or a high initial GCS that decreases over time, are predictive of a poorer outcome than a consistently high GCS or a low initial GCS score that progressively improves with time. It should be noted that the vast majority of MTBI patients have a GCS of 15 at the time of presentation to the ED, indicating no readily apparent CNS injury.[30]

The clinical approach to the diagnosis of MTBI requires a high index of suspicion and should ideally incorporate the use of neurocognitive screening tools. Examples of such tools are the Military Acute Concussion Evaluation (MACE) developed by the DVBIC, and the Standardized Assessment of Concussion (SAC) developed by the 2004 Prague International Conference on Concussion in Sport.[11] In general, these screening tools measure orientation, immediate memory, concentration, and delayed recall.[11] In collaboration with CDC, researchers from the Children's National Medical Center (Washington, DC) and the University of Pittsburgh Medical Center have developed the Acute Concussion Evaluation (ACE) and the Pediatric Acute Concussion Evaluation (PACE). The ACE and PACE are evaluation tools and management guides for use in primary care, sports medicine, and emergency medicine.[51]

The 2002 American College of Emergency Physicians (ACEP) evidence-based clinical policy addressing neuroimaging and decision making in adult MTBI in the acute setting discourages the use of skull radiographs in the evaluation of MTBI because of lack of sensitivity.[30] An updated ACEP evidence-based clinical policy addresses which patients with MTBI should have a noncontrast computed tomography (CT) scan of the head in the ED.[52] The policy guideline differentiates between patients with and without loss of consciousness. This policy includes a level A recommendation that a head CT without contrast is indicated in head trauma patients with loss of consciousness or posttraumatic amnesia only if one or more of the following is present: headache, vomiting, age greater than 60 years, drug or alcohol intoxication, deficits in short-term memory, physical evidence of trauma above the clavicle, posttraumatic seizure, GCS score less than 15, focal neurologic deficit, or coagulopathy. For patients without loss of consciousness or posttraumatic amnesia, a level B recommendation specifies that a noncontrast head CT should be considered if there is one of the following indications: focal neurologic deficit, vomiting, severe headache, age 65 years or greater, physical signs of a basilar skull fracture, GCS score less than 15, coagulopathy, or a dangerous mechanism of injury.[52]

This same 2008 ACEP evidence-based policy document indicated that the scientific literature is not adequate to make any recommendation regarding the role for head MRI in the ED evaluation of a patient with acute MTBI, even though MRI potentially increases the sensitivity for detection of acute traumatic intracranial injury by up to 30% in patients with MTBI.[52] There is a lack of well-designed studies evaluating the use of MRI within 24 h of injury, and there are many potentially limiting factors for using MRI to diagnose MTBI in the ED, including cost constraints, availability, and accessibility. However, recent and future improvements in technology may lead to a role for MRI in ED diagnosis.[52]

There is no readily available biochemical marker currently recommended for clinical use in evaluation for MTBI; however, the cellular mechanisms of injury in the cascade of neurochemical changes that accompany physical injury to the brain, including lipid peroxidation, mitochondrial damage, and apoptosis, lend themselves to definite possibilities for the development of biochemical markers. Potential markers studied thus far include glucose, norepinephrine, epinephrine, and dopamine; the neuronal proteins neuron-specific enolase and tau; and the astrocyte proteins S-100B, creatine kinase BB isoenzyme, and glial fibrillary acidic protein. Though the Food and Drug Administration (FDA) has not yet approved S-100B for clinical use in the United States, ACEP has included a level C recommendation regarding its possible use as an aid to neuroimaging decision making based on European research. The recommendation applies only to MTBI patients without significant extracranial injuries seen within 4 h of injury. In this well-defined group, a serum S-100B level less than 0.1 µg/l may permit the clinician to consider not performing a head CT in the ED.[52]

Survivors who have been in proximity to explosions may also have signs and symptoms of ASD immediately following the event. Symptoms include recurrent images in dreams or flashbacks, extreme anxiety, or a numb or detached affect. When symptoms persist for more than a month, patients may have PTSD. Symptoms can also include hypervigilence, insomnia, and an exaggerated startle response. An important caveat for the clinician is that a diagnosis of ASD or PTSD does not exclude the possibility of an underlying MTBI. Treatment options and outcome depend upon appropriate diagnosis or combination of diagnoses.

Lack of consensus in definitions and overlapping of the signs and symptoms complicates the diagnosis of concussion, PCS, MTBI, ASD, and PTSD, each of which may occur following blast exposure. An important caveat for the clinician is that a diagnosis of ASD, PCS, or PTSD does not exclude the possibility of an underlying MTBI. For the busy emergency physician providing medical treatment to multiple victims of an explosion, sufficient time to administer one of the neurocognitive screening tools for MTBI may not be available. Although these diagnoses may not be initially made by the emergency physician, admitting physicians as well as patients and family should be made aware of possible MTBI signs and symptoms that could later lead to diagnosis of MTBI. The emergency physician should be prepared to make the diagnosis of post-blast concussion and consider the diagnosis of MTBI in patients with a GCS of 13-15, moderate TBI in patients with GCS 9-12, and severe TBI in patients with GCS 3-8.[8]

One clinical entity unique to the blast-injured patient is arterial air embolism (AAE) secondary to pulmonary parenchymal disruption in blast lung injury. An altered mental status or focal neurological deficit in a patient with blast lung injury, especially following positive-pressure ventilation, should arouse suspicion of AAE.[34]

## TREATMENT

The treatment of open and closed skull fractures, penetrating brain injuries, and subarachnoid, subdural, and epidural hematomas resulting from an explosion is similar to treatment of these conditions resulting from other trauma. Initial treatment should follow established clinical protocols. In the more severely injured patient, exacerbation of brain injuries or development of secondary brain injuries may be related to hypotension and hypoxemia. Aggressive prehospital and ED airway control, ventilation, oxygenation, and fluid therapy as indicated may be required to preserve optimal neurologic status. Routine hyper ventilation of the brain-injured patient is no longer advocated.[53]

One important clinical treatment caveat in the setting of TBI from explosions is in the diagnosis and treatment of AAE that results from blast lung injury. The symptoms of AAE may not begin until the onset of positive-pressure ventilation, so an awareness of this possibility is important for differential diagnosis and treatment of altered mental status in the bomb blast victim. Fowler positioning for shock may increase the likelihood of cerebral AAE. To prevent air embolism, the patient should instead be positioned in such a way that the coronary artery ostia lies in their lowest positions. The prone or semi-left lateral (more towards prone than classic left lateral) positioning is recommended. The treatment of choice for AAE is hyperbaric oxygen therapy.[34]

Some authors suggest that physical activity following the event may also increase the likelihood of AAE and other adverse sequelae of blast injury. Physical activity of victims exposed to blast waves should therefore be limited in the field.[34] Bomb blast victims with possible brain injury should be advised to rest and refrain from strenuous physical activity, such as aiding other victims of the blast.

Treatment of concussion injuries includes providing the patient and family with educational materials and observation guidelines to determine any deterioration of function after discharge. Two randomized clinical trials have shown the importance of patient education and follow-up in reducing the number and severity of post-concussive symptoms.[54,55] Education should include the fact that symptoms completely resolve within 7-10 days in approximately 90% of individuals who sustain a concussion. However, information should also be provided indicating the possibility of persistence of symptoms in a small minority of individuals, and the need for appropriate primary care or neurology follow-up for those individuals.

A period of relative rest for the first 2-5 days following concussion is generally recommended, with a stepwise increase in physical activity. Acetaminophen is generally recommended for headaches in these individuals.[8] In addition, other pharmacologic interventions may be used to treat specific symptoms following MTBI. Headaches may be treated with nonsteroidal anti-inflammatory agents, isometheptene, tryptans, beta-blockers, tricyclic antidepressants, or divalproex. Secondary depression may improve with selective serotonin-releasing inhibitors. Meclizine may be helpful for dizziness or vertigo. Nonpharmacologic interventions such as relaxation techniques may be helpful, and cognitive-behavioral interventions may be considered.[56] ACEP is currently developing evidence-based recommendations for ED discharge instructions following MTBI as a follow-on to the 008 neuroimaging and decision-making guidelines with the hope of having better standardization of ED discharge instructions.[52]

## CONCLUSIONS AND RECOMMENDATIONS

The potential for major terrorist bombing attacks in the United States is a reality for which acute care clinicians must prepare. Terrorist bombing is a more likely scenario than an attack with chemical or biological weapons. TBI is an important cause of morbidity and mortality following a high-order explosive event. The severity and nature of brain injuries that occur depend partly upon the nature and quantity of the explosive used. Severity of injury is markedly increased for victims within an enclosed space, and severity is inversely proportional to the cube of the distance between the victim and the epicenter of the event.

Both open and closed brain injuries can occur as a result of a blast, and include penetrating brain injury, skull fracture, diffuse axonal injury, cerebral contusion, and epidural and subdural hemorrhage. AAE of the brain can also occur as a consequence of blast lung injury, and can be precipitated by positive-pressure ventilation. Concussion, PCS, MTBI, ASD, and PTSD can also occur as a result of exposure to blast events.

Diagnosis and treatment for TBIs that occur as a result of explosion is similar to that for brain injury that occurs as a result of other traumas. Emergency physicians should always maintain an awareness that concussion and MTBI can occur in blast victims. These conditions could go undiagnosed in the ED due to time and resource constraints imposed by multicasualty bombing events. Patients admitted to the hospital for other injuries should be screened for these conditions before discharge. Patients should be provided appropriate educational materials and referral upon discharge in the event that new signs and symptoms emerge, or if existing symptoms worsen or fail to resolve.

Improvement in treatment and outcome for patients with MTBI resulting from a blast event can only occur if this entity is recognized. Enhanced recognition can be achieved through greater awareness and pre-event education. Further clarifications and consensus among experts on definitions and diagnostic criteria will increase the likelihood of accurate diagnoses of concussion, PCS, MTBI, ASD, and PTSD. Further research and development in the fields of neuroimaging and biochemical markers should improve the ability to diagnose TBI secondary to blast events. Well-designed studies of current and future treatment modalities are essential for evidence-based care recommendations following TBI resulting from a blast event.

## References

[1] Born CT (2005). Blast trauma: the fourth weapon of mass destruction. Scand J Surg.

[2] Frykberg ER (2002). Medical management of disasters and mass casualties from terrorist bombings: how can we cope?. J Trauma.

[3] Kapur GB, Hutson HR, Davis MA, Rice PL (2005). The United States twenty-year experience with bombing incidents: implications for terrorism preparedness and medical response. J Trauma.

[4] de Ceballos JP, Turegano-Fuentes F, Perez-Diaz D, Sanz-Sanchez M, Martin-Llorente C, Guerrero-Sanz JE (2005). 11 March 2004: The terrorist bomb explosions in Madrid, Spain--an analysis of the logistics, injuries sustained and clinical management of casualties treated at the closest hospital. Crit Care.

[5] Frykberg ER, Tepas JJ 3rd (1988). Terrorist bombings. Lessons learned from Belfast to Beirut. Ann Surg.

[6] Zouris JM, Walker GJ, Dye J, Galarneau M (2006). Wounding patterns for U.S. marines and sailors during Operation Iraqi Freedom, major combat phase. Mil Med.

[7] Owens BD, Kragh JF, Wenke JF, Macaitis JC, Wade CE, Holcomb JB (2008). Combat wounds in Operation Iraqi Freedom and operation enduring freedom. J Trauma.

[8] Willer B, Leddy JJ (2006). Management of concussion and post-concussion syndrome. Curr Treat Options Neurol.

[9] Kay T, Harrington D, Adams R, Anderson R, Berrol S, Cicerone K (1993). Definition of mild traumatic brain injury. J Head Trauma Rehabil.

[10] US Dept of Health and Human Services. (2003). Centers for Disease Control and Prevention. Report to Congress on mild traumatic brain injury in the United States: steps to prevent a serious public health problem.

[11] Walter Reed Army Medical Center. Defense and Veterans Brain Injury Center Working Group. Consensus conference on the on the acute management of concussion/mild traumatic brain injury (mTBI) in deployed settings (2008). http://www.dvbic.org/images/pdfs/Providers/Deployed_setting_CPG_10OCT08.aspx.

[12] American Academy of Neurology Quality Standards Subcommittee (1997). Practice Parameter: the management of concussion in sports (summary statement). Neurology.

[13] Ropper AH, Gorson KC (2007). Clinical practice. Concussion. N Engl J Med.

[14] Ryan LM, Warden DL (2003). Post concussion syndrome. Int Rev Psychiatry.

[15] Jones E (2006). Historical approaches to post-combat disorders. Philos Trans R Soc Lond B Biol Sci.

[16] American Psychiatric Association (2000). Task Force on DSM-IV. Diagnostic and statistical manual of mental disorders: DSM-IV-TR.

[17] Trudeau DL, Anderson J, Hansen LM, Shagalov DN, Schmoller J, Nugent S (1998). Findings of mild traumatic brain injury in combat veterans with PTSD and a history of blast concussion. J Neuropsychiatry Clin Neurosci.

[18] Cernak I, Savic J, Ignjatovic D, Jevtic M (1999). Blast injury from explosive munitions. J Trauma.

[19] Great Britain. War Office (1922). Committee of Enquiry into ‘Shell-Shock’. Report of the war committee of enquiry into ‘Shell-Shock’. Parliament Papers by Command. 1734.

[20] Myers CS (1915). A contribution to the study of shell shock: being an account of three cases of loss of memory, vision, smell, and taste admitted into the Duchess of Westminister's War Hospital, Le Touquet. Lancet.

[21] Macleod AD (2004). Shell shock, Gordon Holmes and the Great War. J R Soc Med.

[22] Mott FW (1916). The Lettsomian lectures on the effects of high explosives upon the central nervous system. Lancet.

[23] Fabing HD (1947). Cerebral blast syndrome in combat soldiers. Arch Neurol Psychiatry (Chicago).

[24] Cramer F, Paster S, Stephenson C (1948). Cerebral injuries due to explosion waves: blast concussion. Arch Neurol Psychiatry.

[25] Mallonee S, Shariat S, Stennies G, Waxweiler R, Hogan D, Jordan F (1996). Physical injuries and fatalities resulting from the Oklahoma City bombing. JAMA.

[26] Helling ER, Pfannenstiel TJ (2005). Comprehensive head and neck trauma screening: the USS Cole experience. Mil Med.

[27] Warden D (2006). Military TBI during the Iraq and Afghanistan wars. J Head Trauma Rehabil.

[28] Lew HL, Poole JH, Alvarez S, Moore W (2005). Soldiers with occult traumatic brain injury. Am J Phys Med Rehabil.

[29] Nelson TJ, Wall DB, Stedje-Larsen ET, Clark RT, Chambers LW, Bohman HR (2006). Predictors of mortality in close proximity blast injuries during Operation Iraqi freedom. J Am Coll Surg.

[30] Jagoda AS, Cantrill SV, Wears RL, Valadka A, Gallagher EJ, Gottesfeld SH (2002). Clinical policy: neuroimaging and decisionmaking in adult mild traumatic brain injury in the acute setting. Ann Emerg Med.

[31] Stahura B (2006). The silent injury comes home from war [Internet]. Reprint by the author. Originally published in the year in veterans affairs and military medicine 2005-2006. http://www.barbarastahura.com/silentinjurycomeshome.html.

[32] Rutland-Brown W, Langlois JA, Nicaj L, Thomas RG, Wilt SA, Bazarian JJ (2007). Traumatic brain injuries after mass-casualty incidents: lessons from the 11 September 2001 World Trade Center attacks. Prehosp Disaster Med.

[33] Taber KH, Warden DL, Hurley RA (2006). Blast-related traumatic brain injury: what is known?. J Neuropsychiatry Clin Neurosci.

[34] Wightman JM, Gladish SL (2001). Explosions and blast injuries. Ann Emerg Med.

[35] Katz E, Ofek B, Adler J, Abramowitz HB, Krausz MM (1989). Primary blast injury after a bomb explosion in a civilian bus. Ann Surg.

[36] Sasser SM, Sattin RW, Hunt RC, Krohmer J (2006). Blast lung injury. Prehosp Emerg Care.

[37] Scott SG, Vanderploeg RD, Belanger HG, Scholten JD (2005). Blast injuries: evaluating and treating the postacute sequelae. Fed Pract.

[38] DePalma RG, Burris DG, Champion HR, Hodgson MJ (2005). Blast injuries. N Engl J Med.

[39] Kluger Y, Nimrod A, Biderman P, Mayo A, Sorkin P (2007). The quinary pattern of blast injury. Am J Disaster Med.

[40] Guy RJ, Glover MA, Cripps NP (2000). Primary blast injury: pathophysiology and implications for treatment. Part III: Injury to the central nervous system and the limbs. J R Nav Med Serv.

[41] Clemedson CJ, Pettersson H (1955). Propagation of a high explosive air shock wave through different parts of an animal body. Am J Physiol.

[42] Hull JB (1992). Traumatic amputation by explosive blast: pattern of injury in survivors. Br J Surg.

[43] Hull JB, Cooper GJ (1996). Pattern and mechanism of traumatic amputation by explosive blast. J Trauma.

[44] Lettieri CJ (2009). Neurotrauma: management of acute head injuries. Medscape Today [Internet].

[45] Cernak I, Savic J, Malicevic Z, Zunic G, Radosevic P, Ivanovic I (1996). Involvement of the central nervous system in the general response to pulmonary blast injury. J Trauma.

[46] Cernak I, Wang Z, Jiang J, Bian X, Savic J (2001). Ultrastructural and functional characteristics of blast injury-induced neurotrauma. J Trauma.

[47] Kaur C, Singh J, Lim MK, Ng BL, Yap EP, Ling EA (1995). The response of neurons and microglia to blast injury in the rat brain. Neuropathol Appl Neurobiol.

[48] Kaur C, Singh J, Lim MK, Ng BL, Ling EA (1997). Macrophages/microglia as'sensors' of injury in the pineal gland of rats following a non-penetrative blast. Neurosci Res.

[49] Moochhala SM, Md S, Lu J, Teng CH, Greengrass C (2004). Neuroprotective role of aminoguanidine in behavioral changes after blast injury. J Trauma.

[50] Teasdale G, Jennett B (1974). Assessment of coma and impaired consciousness. Apractical scale. Lancet.

[51] US Dept of Health and Human Services (2007). Centers for Disease Control and Prevention Internet].'Heads Up': brain injury in your practice. http://www.cdc.gov/TraumaticBrainInjury/physicians_tool_kit.html.

[52] Jagoda AS, Bazarian JJ, Bruns JJ, Cantrill SV, Gean AD, Howard PK (2008). Clinical policy: neuroimaging and decisionmaking in adult mild traumatic brain injury in the acute setting. Ann Emerg Med.

[53] American College of Surgeons Committee on Trauma (2004). ATLS: advanced trauma life support, program for doctors.

[54] Ponsford J, Willmott C, Rothwell A, Cameron P, Kelly AM, Nelms R (2002). Impact of early intervention on outcome following mild head injury in adults. J Neurol Neurosurg Psychiatry.

[55] Wade DT, King NS, Wenden FJ, Crawford S, Caldwell FE (1998). Routine follow up after head injury: a second randomized controlled trial. J Neurol Neurosurg Psychiatry.

[56] Warden D, Helmick K, Ryan L, Sparling M, Stevens AM http://www.cdc.gov/TraumaticBrainInjury/physicians_tool_kit.html.

